# Smoking a Dangerous Addiction: A Systematic Review on an Underrated Risk Factor for Oral Diseases

**DOI:** 10.3390/ijerph182111003

**Published:** 2021-10-20

**Authors:** Naveed Ahmed, Sohaib Arshad, Syed Nahid Basheer, Mohmed Isaqali Karobari, Anand Marya, Charu Mohan Marya, Pratibha Taneja, Pietro Messina, Chan Yean Yean, Giuseppe Alessandro Scardina

**Affiliations:** 1 Department of Medical Microbiology and Parasitology, School of Medical Sciences, Universiti Sains Malaysia, Kubang Kerian, Kota Bharu 16150, Malaysia; namalik288@gmail.com; 2 Periodontics Unit, School of Dental Sciences, Health Campus, Universiti Sains Malaysia, Kubang Kerian, Kota Bharu 16150, Malaysia; arshadsohaib993@gmail.com; 3 Department of Restorative Dental Sciences, College of Dentistry, Jazan University, Jazan 45142, Saudi Arabia; snbasheer@jazanu.edu.sa; 4 Conservative Dentistry Unit, School of Dental Sciences, Health Campus, Universiti Sains Malaysia, Kubang Kerian, Kota Bharu 16150, Malaysia; 5 Department of Conservative Dentistry & Endodontics, Saveetha Dental College & Hospitals, Saveetha Institute of Medical and Technical Sciences University, Chennai 600077, India; 6 Department of Orthodontics, University of Puthisastra, Phnom Penh 12211, Cambodia; amarya@puthisastra.edu.kh; 7 Department of Orthodontics, Saveetha Dental College & Hospitals, Saveetha Institute of Medical and Technical Sciences University, Chennai 600077, India; 8 Department of Public Health Dentistry, Sudha Rustagi College of Dental Sciences and Research, Faridabad 121002, India; maryacm@yahoo.co.uk (C.M.M.); pratibhataneja3@gmail.com (P.T.); 9 Department of Surgical, Oncological and Stomatological Disciplines, University of Palermo, 90133 Palermo, Italy; pietro.messina01@unipa.it

**Keywords:** oral health, smoking, periodontal disease, risk factors, oral cancer

## Abstract

Despite growing knowledge of the adverse effects of cigarette smoking on general health, smoking is one of the most widely prevalent addictions around the world. Globally, about 1.1 billion smokers and over 8 million people die each year because of cigarette smoking. Smoking acts as a source for a variety of oral and systemic diseases. Various periodontal issues such as increased pocket depth, loss of alveolar bone, tooth mobility, oral lesions, ulcerations, halitosis, and stained teeth are more common among smokers. This systematic review was conducted according to the guidelines from PRISMA, and research articles were retrieved from the Web database sources on 31 May 2021. The quality of research articles was ensured by the type of evidence from combined schema incorporating as schema-13 evidence type description, Cochrane health promotion and public health field (CHPPHF), and the health gains notation framework-14 screening question for quality assessment of qualitative and quantitative studies. Smokers have been found to have bleeding on probing, periodontal pockets, and clinical attachment loss compared to nonsmokers. Oral and respiratory cancers are among the most lethal known diseases caused by cigarette smoking and other commonly occurring sequelae such as stained teeth, periodontal diseases, etc.

## 1. Introduction

Oral diseases appear to be a global problem that should be addressed as a matter of global health concern. Oral health issues include various behavioral and social features such as habits, oral health knowledge, practices, availability, modifiable risk factors, and accessibility to oral health treatments [1]. Health is considered as a significant factor in making life valuable [2]. In general, lifestyles and behavioral patterns are continuously changing, making people more susceptible to oral disorders. Common preventable risk factors for oral diseases include consuming great amounts of sugary food and alcohol and smoking excessively [3]. Back in 2015, untreated oral disorders crippled over half of the world’s population (age-standardized prevalence: 48.0 percent), affecting 3.5 million individuals worldwide [4,5].

Oral and orofacial problems can affect children and adolescents, affecting physical functioning and psychosocial well-being [6]. One of the scientific theories used to influence human health-related behaviors is the knowledge, attitude, and practices (KAP) theory. According to the KAP theory, healthy knowledge is the foundation for developing an optimistic and healthy lifestyle, attitudes are the motivating factor behind changing behavior, and the goal is to promote oral health [7]. This is why oral health professionals play a critical role in disease prevention and diagnosis through screening and raising awareness [8]. Recently, a shift of focus in health care has been noticed, signaling a transition from biological to a more complete and broader biopsychosocial concept of health [9].

The oral cavity is a speculum for a person’s current health issues. Some of the modifiable risk factors for poor oral hygiene include cigarette smoking, betel quid chewing, and alcohol consumption. Despite the fact that there is growing knowledge of the adverse effects of cigarette smoking on general health, smoking is one of the widely prevalent addictions around the world [10]. Globally, about 1.1 billion smokers and over 8 million people die each year due to cigarette smoking [11]. Smoking acts as a source for a variety of diseases, including cardiovascular diseases (CVD), chronic obstructive pulmonary diseases (COPD), cancer, and periodontal disease (POD), as one of the five top risk factors for the global burden of the disease [12,13,14]. According to the alcohol and drugs survey, 15% of people currently smoke cigarettes, with 17% of men and 13% of women. Teenagers aged 15–19 years have been found to smoke at an estimated rate of 8%, with 10% of males and 6% of females being current smokers. The frequency was 16 percent among people aged 20–24 years and 25 years and older [15].

**Figure 1:**
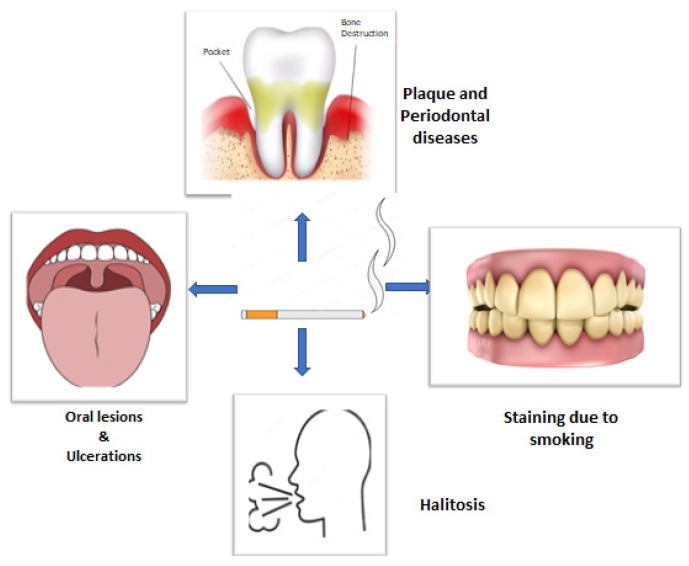
Diagrammatic presentation of possible effects of smoking on oral health.

Tobacco smoking has numerous and well-documented negative consequences. The oral cavity is the first to get exposed to cigarette smoke, wherein the soft and hard tissues come in direct contact, making it the first area of confrontation [16]. Tobacco smoking, particularly in the form of cigarettes, has been proved to be a significant risk factor for periodontitis (Figure 1) [17]. Other than plaque, smoking has been identified as an important risk factor for POD. It also affects the prevalence of POD, severity, progression, and treatment response. According to epidemiological research, smokers have a much higher risk of POD than nonsmokers, and the increased risk is proportionate to the duration and rate of smoking [18,19]. Various gingival and periodontal issues such as gingivitis, increased pocket depth, loss of alveolar bone, tooth mobility, oral lesions, ulcerations, halitosis, and stained teeth are more common among smokers [20].

According to a meta-analysis, exposure to cigarette smoke in the environment relates to a considerably increased risk of lung cancer [21]. Cigarette smoking has also been linked to several other oral cancers. Kumar, A. et al. presented a clinic-pathological investigation that showed that 29.4% of people with established oral cancer cases chewed only tobacco, 25.5% only smoked, 42.2% chewed both types of tobacco (smoke and nonsmokers), and 2.9% did not chew tobacco. 83.3% of those who solely chewed tobacco had oral cavity malignancies, with 6.7% having malignancies of the oropharynx and hypopharynx. Of those who only smoked tobacco, 69.2% of individuals had the disease [22]. This predicts that there is a high chance of developing cancer regardless of how you use tobacco (smoking, chewing, etc.).

Rationale: The most critical risk factor associated with the onset of various gingival and periodontal diseases is tobacco smoking. It reduces the quality of life of patients and poses a risk to oral health. It has been demonstrated that oral health among smokers is compromised in comparison to nonsmokers. Thus, this study is aimed at reviewing the literature to evaluate the effect of smoking on oral health.

Objectives: In this systematic review, we aim to examine the effects of cigarette smoking on oral health, present the major oral diseases caused by cigarette smoking, and determine if there is any possibility of bacterial or fungal infections among smokers.

## 2. Material and Methods

### 2.1. Protocol and Registration

This systematic review was conducted according to the guidelines from PRISMA (http://www.prisma-statement.org accessed on 4 October 2021). Research articles were retrieved from the Web database sources on 31 May 2021. The study has been registered on PROSPERO with registration number CRD42021273462. Initially, articles were assessed from MDPI, PubMed, Scopus, and Web of Science (WOS).

### 2.2. Research Questions

The research questions of this systematic review are as follows: What are the effects of cigarette smoking on oral health? What are the major oral diseases caused by cigarette smoking? Is there any possibility of bacterial or fungal infections among smokers?

### 2.3. Data Sources

Relevant articles for inclusion in the review were found through a search of electronic databases. Keywords included “smoking and or oral health”, “cigarette smoking”, “Smoking effects and or oral health”, and “smoking and or tobacco use”.

### 2.4. Search Strategies

Electronic databases were searched for articles according to the selected keywords such as smoking, cigarette smoking, and tobacco use following the MeSH strategy, which evaluates the effects of cigarette smoking on oral health, published between 2011 to 2021. The number of articles retrieved from each database is shown in Table 1.

### 2.5. Study Selection and Criteria for Eligibility of Articles

A detailed search of research articles from Web sources was performed to find out the studies that examined the effects of cigarette smoking on oral health and were published between 2011 to 2021. Two researchers assessed possibly relevant research papers against the previously specified inclusion and exclusion criteria to validate the selection approach, as shown in Table 2.

### 2.6. Quality Assessment

The quality of research articles was ensured by the type of evidence from combined schema incorporating as schema-13 evidence type description, Cochrane health promotion and public health field (CHPPHF), and the health gains notation framework-14 screening question for quality assessment of qualitative and quantitative studies.

The CHPPHF quality assessment tool was utilized to assess the quality of articles. This instrument scored the criteria for allocation bias, selection bias, intervention integrity, blinding, withdrawals and dropouts, confounding, data collecting procedures, and statistical analysis for internal and external validity.

Qualitative research was evaluated and scored for quality using questions adapted from the CHPPHF’s Critical Appraisal Skills Programme. There were 19 Type V evidence articles among 3696 articles that have not been further rated for quality. The studies were categorized as weak, moderate, or strong evidence based on their quality.

The quality of published evidence was categorized as I, II-1, II-2, II-3, and III. The articles that had at least evidence from the one proper RCT were categorized as “I”. Articles with data from well-designed controlled trials that were not randomized were classed as “II-1”. Evidence from well-designed case-control analytic investigations or cohort, ideally by many centers or research groups, was graded as “II-2”. This included evidence from time or place comparisons with or without the intervention. “II-3” was used to describe dramatic results from uncontrolled studies. While renowned experts’ judgments were based on clinical experience, descriptive research or expert committee reports were designated as “III.”

A, B, C, D, and E were the categorized grades based on recommendations. Research having sufficient evidence to support the suggestion that the condition is included in a periodic health examination (PHE) were categorized as “A”. Reports with sufficient evidence to recommend that the condition be specially examined in a PHE were categorized as “B”. Reports that were given the “C” grade had too little evidence to support the inclusion or exclusion of a disease from a PHE; recommendations may be made on other reasons. Reports with sufficient evidence to indicate that a condition is expressly omitted from consideration in a PHE were categorized as “D”. When there was adequate information found to support the suggestion that the condition is expressly excluded from PHE, it was considered as “E”.

### 2.7. Data Extraction

Two researchers (S.A. and N.A.) performed the independent sampling and extraction of required data from the papers included in the current analysis after reading the entire text. The authors of the study, year of publication, study duration, business, group of references, country, number of samples, type of samples, and smoking effects on oral health were all extracted and reported in Table 3.

## 3. Results

### 3.1. Study Selection Results

**Figure 2:**
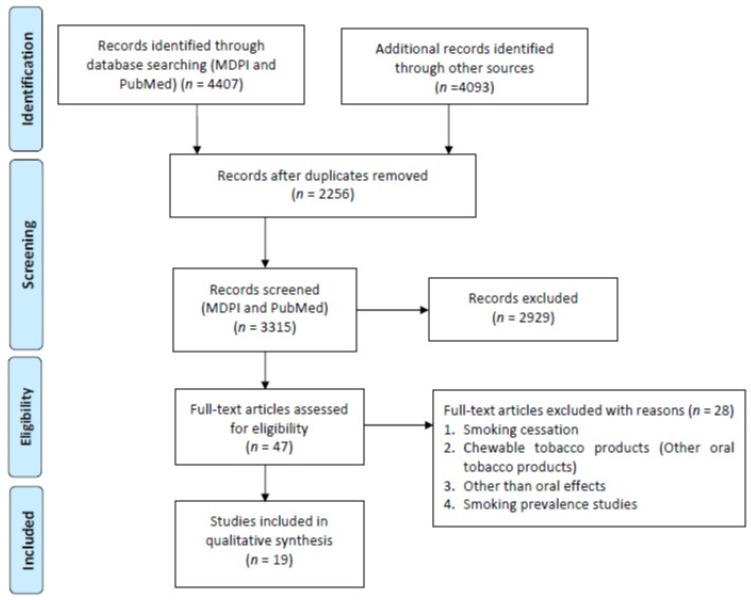
Flowchart of articles retrieved from different Web sources.

**Figure 3:**
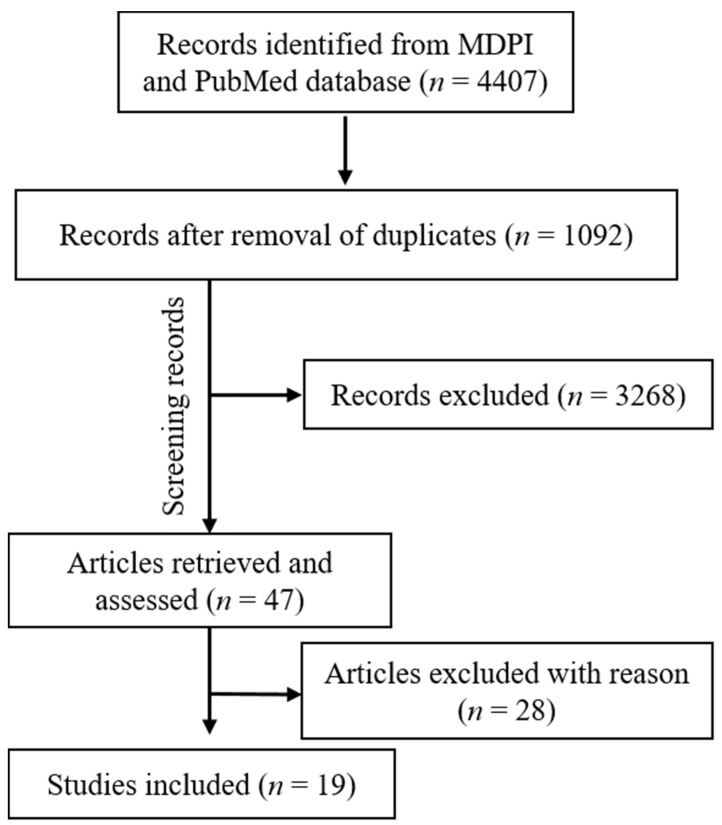
Articles included in the current study (MDPI and PubMed).

A total of 3696 studies were retrieved from the PubMed, Scopus (2271), WOS (1822), and MDPI (711) according to the inclusion/exclusion criteria as listed in Table 2. Data were extracted from 19 studies that purely met the eligibility criteria. Figure 2 and Figure 3 shows the results of the total studies evaluated. Initially, a total of 3696 articles were screened from PubMed, 711 from MDPI and 4093 form other databases (Scopus and WOS) as per the search criteria described in Table 1. After identifying duplicate articles, 972 were excluded from PubMed, 657 from Scopus, 507 from WOS, and 120 from MDPI. After removal of duplicates, the remaining 3315 articles were screened by reading the title and abstracts, after which 2929 articles were excluded. The remaining 47 articles were then fully read and assessed for the inclusion/exclusion criteria and quality assessment. After reading the full text, 28 studies were excluded due to reasons including cigarette cessation studies with no oral effects, prevalence of cigarette smoking among different population reporting no oral effects, questionnaire-based studies, and studies with chewable tobacco or other oral tobacco products. The finally selected articles were finalized to proceed further for data extraction. Based on the quality assessment of the research studies, these 19 articles were screened for the present study. A meta-analysis of the studies included in this study was not performed due to the methodological heterogeneity of the findings.

From the total retrieved articles (MDPI, PubMed, Scopus, and Web of Sciences), only MDPI and PubMed articles were further included in this study.

### 3.2. Study Features

A total of 19 studies were included in this systematic review. The studies were conducted in different countries; many researchers cited different time durations. The number of citations for each of the study was observed from Google Scholar. Each of the included studies was published in reputed and indexed journals. Table 3 summarizes the sample type, total sample size, adopted methodology, results, and the conclusion of each study. Different types of samples were collected from the patients to observe the effects of smoking on oral health, including biopsies, blood, buckle cells, teeth, saliva, etc. In the included studies, a total of 26,236 samples were observed.

### 3.3. Quality Assessment of Research Articles

All of the articles were shortlisted and screened based on the inclusion and exclusion criteria, titles, and abstract. Full texts were read one by one after the screening process and assessed for the quality of material using the CHPPHF recommendations. CHPPHF assessed articles for internal and external validity and rated the criteria for allocation biases, selection biases, intervention integrity, blinding, withdrawals and dropouts, confounding, data collection methods, and statistical analysis. No statistical assessment of publishing bias was carried out for the included studies as there were limited experimental techniques. A total of eight RCTs were included in the present systematic review.

**Figure 4:**
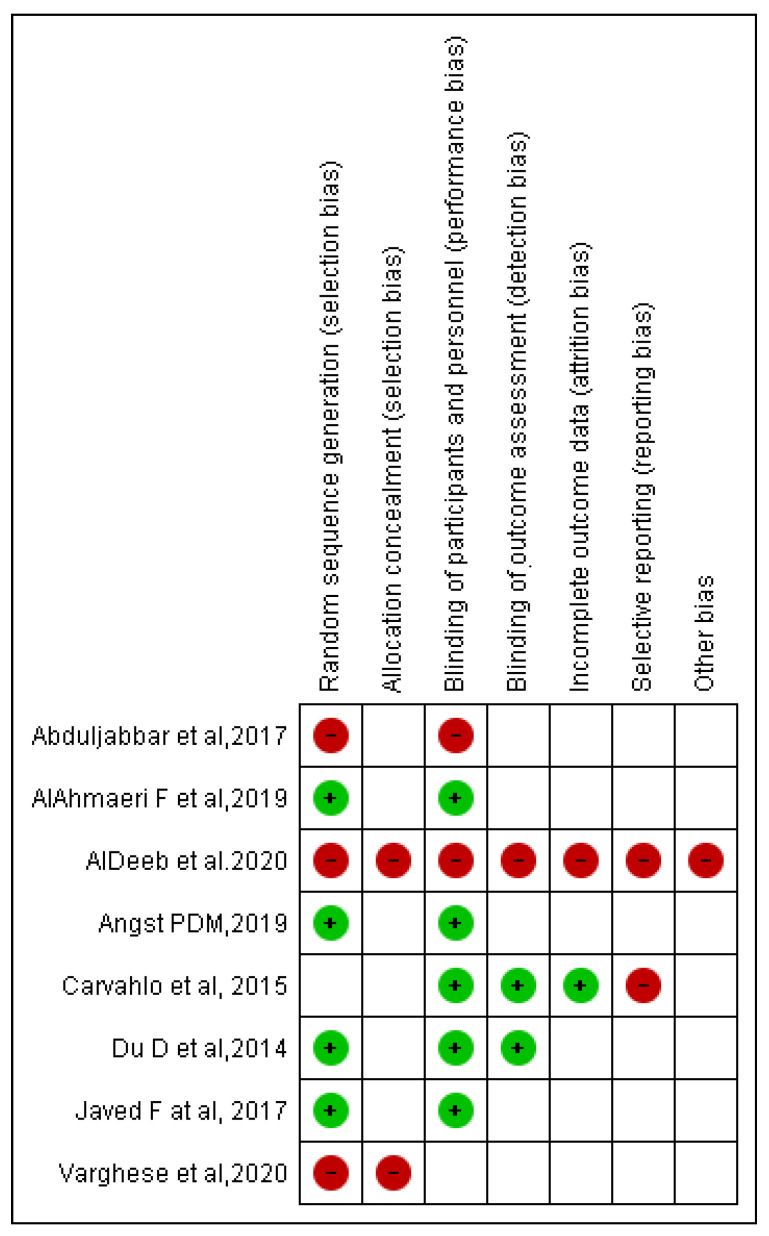
Risk of bias summary: review authors judgment about each risk of bias item for each included RCT.

Bias assessment was conducted according to the Cochrane tool of bias risk assessment. Overall, four included RCTs were at higher risk of bias, four were at lower risk of bias, and many items according to Cochrane tool of bias risk assessment were unclear in eight included RCTs. Selection bias was observed in two of the eight included RCTs, as was performance bias in two studies, detection bias in one study, attrition bias in one study, reporting bias in two studies, and other concerns in one study (Figure 4).

## 4. Discussion

Cigarette smoking has been linked to a variety of health problems. When comparing current smokers to nonsmokers, the rate of mortality from any cause was two to three times higher [42,43]. In many smoking-related studies, the duration of smoking, a quantity of cigarettes smoked per day, brand of cigarettes smoked, cigarette type, and topographical factors related to smoking all are linked to the severity of tobacco consumption [19]. Because of tobacco use, other lifestyle risk factors, and poor dental care usage, smokers are at a higher risk for many oral diseases. As many oral health problems go unrecognized and untreated, the lack of regular dental care becomes particularly problematic [8,44].

Oral diseases are one of the most frequent chronic diseases, and they are significant public health issues due to their prevalence, effect on people and society, and treatment costs [33,45]. Oral disease determinants are well understood. Oral hygiene, smoking, drinking, hazardous behaviors, and stress are all risk factors for various chronic diseases, and efficient public health interventions to prevent oral diseases [7]. Smoking is one of the most common risk factors for oral diseases [3].

Dental caries and periodontal disease and the probable consequences of both (tooth loss) are serious dental public health issues that affect people all over the world [6,21,46]. Individuals’ quality of life and general health are negatively impacted by poor oral health and untreated oral illnesses [9]. A significant positive association between tobacco smoking and higher risk for periodontitis has been found in prospective longitudinal studies.

The elevations in interleukin (IL)-1 and IL-6 associated with smoking levels upregulate bone resorption through the increase in the ratio between the receptor activator of nuclear factor-κβ ligand (RANKL) and its inhibitor osteoprotegerin (OPG). In addition, higher concentrations of elastase and matrix metalloproteinase (MMP)-8 and MMP-9 with proteolytic activity and decreased levels of protease inhibitors, such as alpha-2-macroglobulin and α-1-antitrypsin, may compromise periodontal healing.

If smoking were eliminated in this population, the risk of periodontitis would be reduced by approximately 14% as calculated using the population attributable risk fraction. In underdeveloped countries, the burden of oral diseases is significantly higher [4,47,48]. Among smokers and nonsmokers, in the treatment of chronic periodontitis (CP), Al-Ahmari et al. (2019) checked the effectiveness of scaling & root planning (SRP) with and without the adjunct antimicrobial photodynamic therapy (aPDT). Bleeding on probing (BOP), plaque index (PI), clinical attachment loss (CAL), and probing pocket depth (PD) 4 mm were all assessed at baseline, one month, and three months of follow-up. Smokers and nonsmokers had similar BOP, PI, PD, and clinical AL at the start of the study. PD, PI, and clinical AL were shown to be greater in smokers than nonsmokers after a one-month and three-month follow-up. At the one-month and three-month follow-ups, all nonsmokers’ BOP, PI, clinical AL, and PD were equivalent [34]. In similar research, Al-Bayaty et al. (2013) conducted a study to observe the effects of cigarette smoking on gingival bleeding, to measure the serum haptoglobin, cotinine, and alpha 1-antitrypsin concentrations in Malaysian smokers. BOP levels were determined to be low, whereas PI values were high. Smokers had considerably more significant levels of serum haptoglobin, cotinine, and alpha 1-antitrypsin than nonsmokers. There was a strong connection between PI and smoking duration (years) and blood cotinine levels [24].

Even though tobacco use has decreased in many high-income countries such as the United States and the United Kingdom, it is growing in many low- and middle-income countries [11,35]. According to the World Health Organization (WHO), there are more than 1.1 billion smokers throughout the world, with more than 80% of them residing in low- and middle-income countries [49]. After nonsurgical periodontal treatment, Varghese et al. (2020) studied the salivary 8-hydroxyguanosine (8-OHdG) levels in smokers and nonsmokers with CP. Clinical periodontal markers (PI, GI, PD, and CLI) were assessed at the start of the study. SRP was performed on patients with CPs (CP smokers) and CPns (CP nonsmokers) [40]. In a three-month follow-up period, all of the clinical measures and salivary collections were repeated. At the baseline period, the PI, GI, PD, and CAL values in the CPs and CPns groups were significantly higher as compared to the CHns and CHs groups. At baseline, salivary levels of 8-OHdG were found significantly higher in the CPs group than the other groups. All of the clinical measures in the CP group improved by the follow-up interval at the third month. However, the salivary levels of 8-OHdG in the CP smoker category were still higher values than the CPns [40]. Haswell et al. (2014) analyzed the biomarkers of biological effect (BOBE) and demonstrated the difference between smokers, nonsmokers, and ex-smokers. The levels of biomarkers were compared, and it was seen that there were 27 possible biomarkers evaluated in all, 14 of which were substantially different between smokers and nonsmokers, and 12 of which were able to discriminate between smokers and former smokers, indicating the possibility of reversibility [26].

The maxillary antrum, submandibular region, salivary glands, and tongue are commonly affected by cervicofacial actinomycosis [35]. The mandible is affected in about half of the cases, with the chin (15%), cheek (15%), and submaxillary ramus and angle (15%). The paranasal sinuses, tongue, larynx, middle ear, thyroid gland, and lachrymal pathways are all nonodontogenic orofacial regions that may also be get affected by cervicofacial actinomycosis [22]. Abduljabbar et al. (2017) worked on a project and wanted to see how effective aPDT was at preventing oral fungus colonization in smokers and nonsmokers suffering from denture stomatitis (DS). Among smokers, a statistically significant decrease in the mean fungal CFU/mL was seen at the 3-month follow-up compared to their respective baseline values of CFU/mL. When compared to their individual baseline values, nonsmokers’ mean levels were lower. After a 3-month follow-up, smokers’ fungal CFU/mL levels were statistically substantially higher than nonsmokers [30].

### Study Limitations

In this systematic review, we have searched the data from a limited number of significant Web sources with specific final publication periods. The articles which have been published in any other sources may be overlooked. We have included articles that were published in the English language; as a result, articles which were published in other languages may also be overlooked.

## 5. Conclusions

Cigarette smoking has well-known hazardous effects on oral health and throughout the respiratory tract. Because of the impairment in oral health, it can also lead to problems in other parts of the body, such as the gastrointestinal tract system. Oral and respiratory cancers are among the most lethal known diseases caused by cigarette smoking, which can also cause plaque, dental caries, and other periodontal diseases. There is also an increased risk for bacterial and fungal infections in the oral cavity. This conclusion is based on a limited number of research studies.

## Figures and Tables

**Table 1:** Search strategy for study-related articles.

Serial #	Keywords	PubMed	Scopus	WOS	MDPI
1	Smoking and oral health	184	910	777	36
2	Smoking effects on oral health	134	223	140	05
3	Cigarette smoking	1186	460	508	421
4	Smoking and tobacco use	2192	678	397	249
Total articles retrieved		3696	2271	1822	711
Removal of duplicate articles		972	657	507	120

**Table 2:** Criteria for inclusion and exclusion of research studies.

Characteristics	Inclusion Criteria	Exclusion Criteria
Study design	Clinical studies including trials and randomized clinical trials, evaluation studies, original research articles	Review articles, narrative reviews, short communication, case report, editorials, letters, unpublished articles, abstracts, systematic reviews, and meta-analyses
Study period	Last 10 years	Articles before 2011.
Publication language	English	Other than English
Interventions	Articles examining the impact of cigarette smoking on oral health initiatives. Individual and community-based studies reported with context, effects, disease outcome of smoking and the process	Articles that only have the observational data.
Publication status	Published	Not published yet

**Table 3:** Methodology, outcomes, and conclusion of articles included in the current study.

Authors	Study Design	Duration of Study	Sample Obtained From	Sample Size	Methodology	Study Outcomes	Author Conclusion
Yuki D et al., 2013 [23]	Quasirandomized cross-over	4 days	Blood, Saliva	16	After smoking one cigarette, the analysis of plasma and saliva nicotine and cotinine.	The concentration profiles of saliva cotinine were identical to those of plasma cotinine, and all of the computed cotinine pharmacokinetic characteristics were the same in both plasma and saliva.	Saliva cotinine, which reflects plasma cotinine content and kinetics, might be a suitable and less intrusive cigarette smoke exposure measurement.
Al-Bayaty et al., 2013 [24]	Comparative cross-sectional	Not mentioned	Blood and teeth	197	BOP, PI, and the levels of serum haptoglobin, cotinine, and alpha 1-antitrypsin were tested.	BOP levels were determined to be low, while plaque indices were modest. Smokers had considerably greater levels of serum haptoglobin, cotinine, and alpha 1-antitrypsin than nonsmokers.	A study found that smoking for a longer period, but not for a higher number of cigarettes per day, was linked to less gingival bleeding in smokers.
Medina Solis CE et al., 2014 [25]	Cross-sectional	Not mentioned	Questionnaire	22,229	Questionnaires base study to evaluate risk factors.	The prevalence of oral or dental diseases was 25.7%.	Tobacco users were more likely to report oral and dental issues.
Haswell et al., 2014 [26]	Cross-sectional	28 days	Urine, Saliva, Blood	263	The levels of biomarkers in 143 smokers, 61 never-smokers, and 61 ex-smokers were compared. A total of 27 potential biomarkers were evaluated.	14 biomarkers were substantially different between smokers and never-smokers, and 12 of these 14 biomarkers could differentiate between smokers and former smokers, indicating the possibility of reversibility.	Twelve of the twenty-seven BOBE are potentially valuable instruments for future product evaluation.
Du D et al., 2014 [27]	RCT	Pre-/postintervention data	Teeth	322	A 0–100 mm visual analog scale was used to measure baseline cravings. Craving assessments were taken at 50 s, 3, 5, 7, 15, 20, 25, and 30 min after the medication was given.	Both treatments demonstrated equal maximal effects on desire alleviation and decreased cue-induced craving. At 50 s, 3 min, and 5 min after treatment, the 2.5 mg nicotine film alleviated cue-induced desire to a larger extent than the 2 mg nicotine lozenge.	While both had equal maximal effects, the 2.5 mg nicotine film alleviated cue-provoked cravings substantially faster than the 2 mg nicotine lozenge. For low-dependence smokers, nicotine film might be effective for providing fast desire relief.
Lee CP et al., 2015 [28]	Case control	5 year	Blood	507	Single-nucleotide polymorphisms (SNPs) in DNA repair genes (CHAF1A and CHAF1B) and a chromosomal segregation gene (AURKA) were discovered using a genotyping assay and gene–environment interaction analysis.	AURKA’s Phe31Ile polymorphism (rs2273535, T91A) was shown a linkage of an increased risk of oral cancer. The 91A allele’s gene dosage was likewise linked to greater risk of mouth cancer. Furthermore, by high usage of cigarettes, the AURKA 91AA homozygote can be modify resulting to a high risk of oral cancer.	The functional Phe31Ile polymorphism of AURKA gene may be a strong susceptibility gene in the incidence of oral cancer.
De Carvalho LD et al., 2014 [29]	RCT	One year	Physical check-up	26	G1: etch-and-rinse in nonsmokers. G2: selective enamel etching in nonsmokers. G3: etch-and-rinse in smokers. G4: selective enamel etching in smokers. One operator applied a nanofilled resin composite to each group and light-cured it sequentially.	Only minor discoloration revealed a statistically significant difference between groups 1, 3, and 4 after 12 months when compared to baseline in the evaluations.	The clinical efficacy of resin composite cervical restorations was unaffected by cigarette smoking.
Abduljabbar T et al., 2017 [30]	RCT	3 months	Oral BBL swab	22	The presence of oral erythematous lesions and the position of the denture in jaws were examined by a clinical oral examination. Exfoliative cytology was used to confirm the appearance of fungal hyphae.	Fungal CFU/mL levels were statistically substantially greater in smokers than nonsmokers at the 3-month follow-up.	In both smokers and nonsmokers, aPDT is effective at inactivating oral fungus colonization.
Javed F et al., 2017 [31]	RCT	12 weeks	Questionnaire/Teeth	54	At baseline and during a 12-week follow-up, periodontal parameters were measured. Group A: mechanical curettage (MC) with adjunct antimicrobial photodynamic therapy (aPDT) Group B: mechanical curettage (MC) only.	Patients in groups A and B had significantly lower PI and PD at 12-week follow-up as compared to their baseline levels. At the 12-week follow-up, patients in Group B had substantially greater PI and PD than those in Group A.	When compared to MC alone, MC with aPDT is more successful in treating peri-implant mucositis in cigarette smokers.
Rodriguez-Rabassa M et al., 2018 [32]	Cross-sectional	Not mentioned	Saliva	34	To evaluate the response from the host, cytokine and chemokine expression analyses were performed.	Some bacterial species connected with the smokers group have associations with hormones and cytokines found to be statistically different between smokers and nonsmokers. Inflammation and carcinogenesis in the oral cavity have been linked to these variables.	The findings might help researchers figure out how the salivary microbiome, host inflammatory responses, and metabolism interacts in smokers.
Blasi PR et al., 2018 [33]	Secondary analysis	2015–2017	Questionnaire	718	Between the baseline and the 6-month follow-up survey, professional dental treatment was received.	The findings provide light on variables that may encourage or discourage low-income smokers from seeking professional dental treatment.	This study provides enough material regarding smoking effects on oral health and motivates the public to visit the dental clinics.
AlAhmari et al., 2019 [34]	RCT	3 months	Teeth	83	The periodontal parameters were examined at baseline, 1 month, and 3 months follow-up.	Probing pocket depth (PD), plaque index (PI), and the clinical AL were all increased in smokers than nonsmokers after 1 and 3 months of follow-up.	For treatment of CP among the cigarette smokers, the results of SRP with and without aPDT are compromised.
Angst PDM et al., 2019 [35]	RCT	2 year	Teeth	62	PD, BOP and CAL.	The test group’s mean PPD was higher at the start than the control groups, but the two groups were similar after two years. Significant decreases in PPD and BOP, as well as an increase in CAL, were found with time, with no significant differences between groups.	During the two years of SPT, oral prophylaxis with oral hygiene instructions alone or in conjunction with subgingival instrumentation was able to preserve the previously achieved periodontal state to a comparable level.
AlDeeb M et al., 2013 [36]	RCT	12 weeks	Peri-implant sulcular fluid, radiography	25	The baseline (before therapy) and the 12-week follow-up (after therapy). The ultrasonic scaler and profuse irrigation were used to provide full-mouth disinfection (FMD). A diode laser was used to perform the photodynamic treatment (PDT).	All groups showed statistically significant reductions in PI and PD markers at the baseline examination and after 12 weeks of follow-up. At 12 weeks, BOP among smokers had increased significantly.	In cigarette smokers, PDT with additional mechanical debridement decreased plaque index and probing depth while increasing probing bleeding and lowering proinflammatory indicators.
Julkunen -Iivari A et al., 2020 [37]	Cohort	1985–2015	Teeth	1080	Associations between tobacco products, periodontal health parameters, education level, and death ages.	Tobacco products, as well as a low level of education, are linked to poor periodontal health. Tobacco users who had a lower level of education had greater PI, calculus, and GI scores than nonusers. When compared to nonusers, missing teeth and a lower education level were associated with a significantly greater frequency of deep periodontal pockets.	Tobacco products were found to be hazardous to periodontal health.
Javed F et al., 2020 [38]	Cohort	Not mentioned	Saliva	46	Full-mouth PI, BOP, PD and AL, marginal bone loss (MBL), and missing teeth. Levels of IL-17A and IL-23 in saliva were measured.	Marijuana users, cigarette smokers, and nonsmokers with periodontitis had poorer clinic-radiographic characteristics than periodontally healthy nonsmokers.	The whole salivary immune-inflammatory response may be somewhat poorer in marijuana users compared to heavy cigarette smokers and nonsmokers with periodontitis.
Nettore IC et al., 2020 [39]	Cross-sectional	Oct 2014-Feb 2019	Flavor recognition	348	Oral administration of aqueous aromatic solutions was used to detect 21 distinct chemicals in the test. The total of the correctly detected tastes was used to determine the flavor score (FS).	Cigarette smoking seemed not to influence flavor recognition.	Smoking was not significantly associated with the flavor identification.
Varghese J et al., 2020 [40]	RCT	3 months	Saliva	40	Periodontal parameters were assessed. Saliva samples were collected before start and after the end of treatment to determine the levels of salivary 8-hydroxyguanosine (8-OHdG) using the ELISA method. CPs: Chronic periodontitis in smokers. CPns: CP nonsmokers (CPns). CHS: Clinically healthy subjects in smokers. CHns: CH nonsmokers.	At baseline, the PI, GI, PD, and clinical AL values in the CPs and CPns groups were substantially greater than those in the CHns and CHs groups. The CPs group had considerably greater baseline salivary levels of 8-OHdG than others. CP group showed improvement by the third-month recall interval; however, the CPs still had a greater level of 8-OHdG values than the CPns.	According to this study for detecting periodontal tissue degradation the salivary 8-OHdG levels might be recognized as an oxidative biomarker, which reveals an ongoing periodontal destructive condition in smokers.
Wychowanski P et al., 2021 [41]	Case series	2012–2015	Bone	164	In the maxilla, immediate implants were placed. Implants were placed in the palatal alveolus in the posterior area. Insertion Torque Value (ITV) and two types of equipment were used to assess implant stability: Periotest (PT) and Osstell (ISQ). Cone beam computed tomography images were used to assess minor bone loss.	Smokers had greater PT values at 6 months post-implantation in an aesthetic region compared to those of nonsmokers. At six months after implantation, smokers’ ISQ scores were considerably lower than nonsmokers’ scores. Smokers had greater PT values in the posterior area than nonsmokers on the day of implantation, 6 months after surgery, and 24 months after surgery. On the day of implantation, as well as 6 months later, smokers had lower ISQ levels than nonsmokers. In the aesthetic and posterior areas, smokers had lower ITV measures than nonsmokers.	According to this study, smoking has a deleterious impact on the durability of immediate implants in the maxilla.

## Data Availability

Any type of data used in this systematic review can be assessed upon the request email to the corresponding author.

## References

[1] Swe K.K., Soe A.K., Aung S.H., Soe H.Z. (2021). Effectiveness of oral health education on 8- to 10-year-old school children in rural areas of the Magway Region, Myanmar. BMC Oral Health.

[2] Zhu S., Häussling V., Aspera-Werz R.H., Chen T., Braun B., Weng W., Histing T., Nussler A.K. (2021). Bisphosphonates Reduce Smoking-Induced Osteoporotic-Like Alterations by Regulating RANKL/OPG in an Osteoblast and Osteoclast Co-Culture Model. Int. J. Mol. Sci..

[3] Sharma S., Trivedi H., Sharma V.K., Gupta N. (2016). Behavioral Factors and Periodontal Disease. Eur. J. Pharm. Med. Res..

[4] Peres M.A., Antunes J.L.F., Watt R.G. (2021). The Contribution of Epidemiology to Oral Health Research. Oral Epidemiology.

[5] Zou Y., Wang D.-H., Sakano N., Sato Y., Iwanaga S., Taketa K., Kubo M., Takemoto K., Masatomi C., Inoue K. (2014). Associations of Serum Retinol, α-Tocopherol, and γ-Tocopherol with Biomarkers among Healthy Japanese Men. Int. J. Environ. Res. Public Health.

[6] Zheng S., Zhao L., Ju N., Hua T., Zhang S., Liao S. (2021). Relationship between oral health-related knowledge, attitudes, practice, self-rated oral health and oral health-related quality of life among Chinese college students: A structural equation modeling approach. BMC Oral Health.

[7] Ueno M., Zaitsu T., Ohara S., Wright C., Kawaguchi Y. (2015). Factors influencing perceived oral health of Japanese middle-aged adults. Asia Pac. J. Public Health.

[8] Shah F.Y., Sehrawat P., Bin A. (2020). Smoking and its ramifications relating to oral mucosa. Int. J. Appl. Dent. Sci..

[9] Omara M., Stamm T., Bekes K. (2021). Four-dimensional oral health-related quality of life impact in children: A systematic review. J. Oral Rehabil..

[10] World Health Organization (2015). WHO Global Report on Trends in Prevalence of Tobacco Smoking 2015.

[11] Almasri A., Wisithphrom K., Windsor L.J., Olson B. (2007). Nicotine and lipopolysaccharide affect cytokine expression from gingival fibroblasts. J. Periodontol..

[12] Collaborators G.R.F. (2018). Global, regional, and national comparative risk assessment of 84 behavioural, environmental and occupational, and metabolic risks or clusters of risks for 195 countries and territories, 1990–2017: A systematic analysis for the Global Burden of Disease Study 2017. Lancet.

[13] Zhang X., Oluyomi A., Woodard L., Raza S.A., Adel Fahmideh M., El-Mubasher O., Byun J., Han Y., Amos C.I., Badr H. (2021). Individual-Level Determinants of Lifestyle Behavioral Changes during COVID-19 Lockdown in the United States: Results of an Online Survey. Int. J. Environ. Res. Public Health.

[14] Yun C., Katchko K.M., Schallmo M.S., Jeong S., Yun J., Chen C.H., Weiner J.A., Park C., George A., Stupp S.I. (2018). Aryl Hydrocarbon Receptor Antagonists Mitigate the Effects of Dioxin on Critical Cellular Functions in Differentiating Human Osteoblast-Like Cells. Int. J. Mol. Sci..

[15] Health Canada (2017). Canadian Tobacco, Alcohol and Drugs Survey (CTADS): Summary of Results for 2017.

[16] Zgliczynska M., Szymusik I., Sierocinska A., Bajaka A., Rowniak M., Sochacki-Wojcicka N., Wielgos M., Kosinska-Kaczynska K. (2019). Contraceptive Behaviors in Polish Women Aged 18–35—A Cross-Sectional Study. Int. J. Environ. Res. Public Health.

[17] Do L.G., Slade G.D., Roberts-Thomson K.F., Sanders A.E. (2008). Smoking-attributable periodontal disease in the Australian adult population. J. Clin. Periodontol..

[18] Bergstrom J. (2014). Smoking rate and periodontal disease prevalence: 40-year trends in Sweden 1970–2010. J. Clin. Periodontol..

[19] Eke P.I., Wei L., Thornton-Evans G.O., Borrell L.N., Borgnakke W.S., Dye B., Genco R.J. (2016). Risk indicators for periodontitis in US adults: NHANES 2009 to 2012. J. Periodontol..

[20] Zhu J., Shi F., Xu G., Li N., Li J., He Y., Yu J. (2019). Conventional Cigarette and E-Cigarette Smoking among School Personnel in Shanghai, China: Prevalence and Determinants. Int. J. Environ. Res. Public Health.

[21] Sheng L., Tu J.-W., Tian J.-H., Chen H.-J., Pan C.-L., Zhou R.-Z. (2018). A meta-analysis of the relationship between environmental tobacco smoke and lung cancer risk of nonsmoker in China. Medicine.

[22] Kumar A., Sharma A., Ahlawat B., Sharma S. (2014). Site specific effect of tobacco addiction in upper aerodigestive tract tumors: A retrospective clinicopathological study. Sci. World J..

[23] Yuki D., Kikuchi A., Miura N., Kakehi A., Onozawa M. (2013). Good relationship between saliva cotinine kinetics and plasma cotinine kinetics after smoking one cigarette. Regul. Toxicol. Pharmacol..

[24] Al-Bayaty F.H., Baharuddin N., Abdulla M.A., Ali H.M., Arkilla M.B., ALBayaty M.F. (2013). The influence of cigarette smoking on gingival bleeding and serum concentrations of haptoglobin and alpha 1-antitrypsin. BioMed Res. Int..

[25] Medina-Solís C.E., Pontigo-Loyola A.P., Perez-Campos E., Cruz P.H., Avila-Burgos L., Mendoza-Rodríguez M., Maupomé G. (2014). National Survey of Oral/Dental Conditions Related to Tobacco and Alcohol Use in Mexican Adults. Int. J. Environ. Res. Public Heal..

[26] Haswell L.E., Papadopoulou E., Newland N., Shepperd C.J., Lowe F.J. (2014). A cross-sectional analysis of candidate biomarkers of biological effect in smokers, never-smokers and ex-smokers. Biomarkers.

[27] Du D., Nides M., Borders J., Selmani A., Waverczak W. (2014). Comparison of nicotine oral soluble film and nicotine lozenge on efficacy in relief of smoking cue-provoked acute craving after a single dose of treatment in low dependence smokers. Psychopharmacology.

[28] Lee C.P., Chiang S.L., Lee C.H., Tsai Y.S., Wang Z.H., Hua C.H., Chen Y.C., Tsai E.M., Ko Y.C. (2015). AURKA Phe31Ile polymorphism interacted with use of alcohol, betel quid, and cigarettes at multiplicative risk of oral cancer occurrence. Clin. Oral Investig..

[29] De Carvalho L.D., Gondo R., Lopes G.C. (2015). One-year clinical evaluation of resin composite restorations of noncarious cervical lesions in smokers. J. Adhes. Dent..

[30] Abduljabbar T., Al-Askar M., Baig M.K., AlSowygh Z.H., Kellesarian S.V., Vohra F. (2017). Efficacy of photodynamic therapy in the inactivation of oral fungal colonization among cigarette smokers and non-smokers with denture stomatitis. Photodiagn. Photodyn. Ther..

[31] Javed F., BinShabaib M.S., Alharthi S.S., Qadri T. (2017). Role of mechanical curettage with and without adjunct antimicrobial photodynamic therapy in the treatment of peri-implant mucositis in cigarette smokers: A randomized controlled clinical trial. Photodiagn. Photodyn. Ther..

[32] Rodríguez-Rabassa M., López P., Rodríguez-Santiago R.E., Cases A., Felici M., Sánchez R., Yamamura Y., Rivera-Amill V. (2018). Cigarette Smoking Modulation of Saliva Microbial Composition and Cytokine Levels. Int. J. Environ. Res. Public Health.

[33] Blasi P.R., Krakauer C., Anderson M.L., Nelson J., Bush T., Catz S.L., McClure J.B. (2018). Factors associated with future dental care utilization among low-income smokers overdue for dental visits. BMC Oral Health.

[34] AlAhmari F., Ahmed H.B., Al-Kheraif A.A., Javed F., Akram Z. (2019). Effectiveness of scaling and root planning with and without adjunct antimicrobial photodynamic therapy in the treatment of chronic periodontitis among cigarette-smokers and never-smokers: A randomized controlled clinical trial. Photodiagn. Photodyn. Ther..

[35] Angst P.D.M., Finger Stadler A., Mendez M., Oppermann R.V., van der Velden U., Gomes S.C. (2019). Supportive periodontal therapy in moderate-to-severe periodontitis patients: A two-year randomized clinical trial. J. Clin. Periodontol..

[36] Al Deeb M., Alresayes S., Mokeem S.A., Alhenaki A.M., AlHelal A., Shafqat S.S., Vohra F., Abduljabbar T. (2020). Clinical and immunological peri-implant parameters among cigarette and electronic smoking patients treated with photochemotherapy: A randomized controlled clinical trial. Photodiagn. Photodyn. Ther..

[37] Julkunen-Iivari A., Heikkinen A.M., Räisänen I.T., Ruokonen H., Meurman J.H., Toppila-Salmi S., Söder P., Söder B. (2020). Tobacco Products, Periodontal Health and Education Level: Cohort Study from Sweden. Dent. J..

[38] Javed F., Al-Zawawi A.S., Allemailem K.S., Almatroudi A., Mehmood A., Divakar D.D., Al-Kheraif A.A. (2020). Periodontal Conditions and Whole Salivary IL-17A and-23 Levels among Young Adult Cannabis sativa (Marijuana)-Smokers, Heavy Cigarette-Smokers and Non-Smokers. Int. J. Environ. Res. Public Health.

[39] Nettore I.C., Maione L., Desiderio S., De Nisco E., Franchini F., Palatucci G., Ungaro P., Cantone E., Macchia P.E., Colao A. (2020). Influences of Age, Sex and Smoking Habit on Flavor Recognition in Healthy Population. Int. J. Environ. Res. Public Health.

[40] Varghese J., Bhat V., Chianeh Y.R., Kamath V., Husain N.A.-H., Özcan M. (2020). Salivary 8-hydroxyguanosine levels in smokers and non-smokers with chronic periodontitis. Odontology.

[41] Wychowański P., Starzyńska A., Jereczek-Fossa B.A., Iwanicka-Grzegorek E., Kosewski P., Adamska P., Woliński J. (2021). The Effects of Smoking Cigarettes on Immediate Dental Implant. Stability—A Prospective Case Series Study. Appl. Sci..

[42] Hamrah M.S., Harun-Or-Rashid M., Hirosawa T., Sakamoto J., Hashemi H., Emamian M.H., Shariati M., Fotouhi A. (2013). Smoking and associated factors among the population aged 40-64 in Shahroud, Iran. Asian Pac. J. Cancer Prev..

[43] Hatsukami D.K., Jensen J., Anderson A., Broadbent B., Allen S., Zhang Y., Severson H. (2011). Oral tobacco products: Preference and effects among smokers. Drug Alcohol Depend..

[44] Ramalingam A., Santhanathas T., Shaukat Ali S., Zainalabidin S. (2019). Resveratrol Supplementation Protects against Nicotine-Induced Kidney Injury. Int. J. Environ. Res. Public Health.

[45] Redondo-Flórez L., Fernández-Lucas J., Clemente-Suárez V.J. (2020). Cultural Differences in Stress-Related Psychological, Nutrition, Physical Activity and Oral Health Factors of Professors. Nutrients.

[46] Wackowski O.A., Manderski M.T.B., Lewis M.J., Delnevo C.D. (2019). The impact of smokeless tobacco risk information on smokers’ risk perceptions and use intentions: A news media experiment. Health Commun..

[47] Radaeli A., Nardin M., Azzolina D., Malerba M. (2019). Determinants of Smoking Status in a Sample of Outpatients Afferent to a Tertiary Referral Hospital. Int. J. Environ. Res. Public Health.

[48] Protano C., Manigrasso M., Cammalleri V., Biondi Zoccai G., Frati G., Avino P., Vitali M. (2020). Impact of Electronic Alternatives to Tobacco Cigarettes on Indoor Air Particular Matter Levels. Int. J. Environ. Res. Public Health.

[49] Jeon H.G., Kim G., Jeong H.S., So W.-Y. (2021). Association between Cigarette Smoking and Physical Fitness Level of Korean Adults and the Elderly. Healthcare.

